# Healthcare professional students’ skills in sexual health communication and history taking: inter-rater reliability of standardized patients and faculty ratings in dar es Salaam, tanzania- a cross-sectional study

**DOI:** 10.1186/s12909-024-05607-8

**Published:** 2024-06-05

**Authors:** Gift G. Lukumay, Stella Emmanuel Mushy, Lucy R. Mgopa, Dickson Ally Mkoka, Agnes F. Massae, Dorkasi L. Mwakawanga, B. R. Simon Rosser, Nidhi Kohli, Corissa T. Rohloff, Michael W. Ross, Maria Trent

**Affiliations:** 1 Muhimbili University of Health and Allied Sciences, United Nations Rd, Dar es Salaam, Tanzania; 2 University of Minnesota, 1300 S. 2nd St., Minneapolis, MN 55454 USA; 3 Johns Hopkins University School of Medicine, 200 N. Wolfe Street, #2056, Baltimore, MD 21287 USA

**Keywords:** Standardized patient, Sexual health, History taking

## Abstract

**Background:**

Low- and middle-income countries face a disproportionate impact of sexual health problems compared to high-income countries. To address this situation proper interpersonal communication skills are essential for clinician to gather necessary information during medical history-taking related to sexual health. This study aimed to evaluate the interrater reliability of ratings on sexual health-related interpersonal communication and medical history-taking between SPs and trained HCP faculty for health care professional students.

**Methods:**

We conducted a cross-sectional comparative study to evaluate the interrater reliability of ratings for sexual health-related interpersonal communication and medical history-taking. The data were collected from medical and nursing students at Muhimbili University of Health and Allied Sciences, who interviewed 12 Standardized Patients (SPs) presenting with sexual health issues. The video-recorded interviews rated by SPs, were compared to the one rated by 5 trained Health Care Professional (HCP) faculty members. Inter-rater reliability was evaluated using percent agreement (PA) and kappa statistics (κ).

**Results:**

A total of 412 students (mean age 24) were enrolled in the study to conduct interviews with two SPs presenting with sexual health concerns. For interpersonal communication (IC), the overall median agreement between raters was slight (κ^2^ 0.0095; PA 48.9%) while the overall median agreement for medical sexual history-taking was deemed fair (κ^2^ 0.139; PA 75.02%).

**Conclusion:**

The use of SPs for training and evaluating medical and nursing students in Tanzania is feasible only if they undergo proper training and have sufficient time for practice sessions, along with providing feedback to the students.

## Background

The World Health Organization (WHO) first defined sexual health in 1975, as the integration of the physical, emotional, intellectual, and social aspects of sexual being, in ways that enrich life and improve personality, communication, and love [1]. However, over the last four decades, the definition has undergone several amendments. It now encompasses having a positive and respectful attitude towards sexuality and relationships, and being able to enjoy safe and pleasurable sexual experiences without discrimination, violence, or coercion, going beyond merely being free from illness or dysfunction (WHO 2006). These amendments aim to promote sexual health within communities and among healthcare providers (HCP) [1, 2]. There is evidence indicating increased awareness of sexual heath among both HCP and the general community [3–5]. Despite these updates, many clients still suffer adverse outcomes related to sexual health issues while under the care of an HCP [6–10]. These challenges pose a threat to the achievement of WHO Millennium Development Goals, which includes the reduction of child mortality, improvement of maternal health, and the fight against HIV/AIDS [11]. Additionally, low- and middle-income countries bear a disproportionate burden of sexual health challenges compared to high-income, industrialized countries [12].

HCPs’ ability to appropriately manage sexual health problems begins with thorough medical history-taking during clinical visits [13]. It is important to integrate sexual health-related medical history-taking into routine healthcare, rather than treating it as a separate entity reserved for specialists [14, 15]. Additionally, incorporating best practices for patient-centered interpersonal communication can optimize the patient-HCP dynamic, enhance the patient’s experience, and foster trust during visits [16].

HCPs who avoid discussing patients’ sexual health concerns often do so due to a lack of knowledge about sexual issues and evidence-based management solutions for certain sexual health problems. They also fear offending patients, violating cultural norms, or having inadequate time to address the concern, resulting in unmet needs for clients’ sexual health services [6–8, 10, 17]. Moreover, the sensitive nature of sexual health concerns necessitates that HCPs possess the art and skill to take a medical history that enables patients to openly discuss their sexual health concerns without hesitation [18–21]. Patients who can freely share details about their sexual health problems with their HCPs tend to achieve better treatment outcomes [22, 23], although studies have indicated that patient perspectives may be overlooked during these conversation [24].

When taking a sexual history, an HCP has to make sure that their posture is open, maintain eye contact with the patient, use open-ended questions, allow the patient to talk, summarize and paraphrase what the patient shared, and use jargon-free language [20, 21]. Additionally, HCPs need to be free from common myths about the causes of sexual health concerns and rely on scientific facts while discussing sexual health-related issues with patients [6–8, 10, 25].

For HCPs to effectively manage the sexual health concerns of their patients, training programs should incorporate a formal, comprehensive sexual health curriculum that is uniformly taught and assessed for all health care professional students [26]. Faculty observation of student and patient encounters, with skills assessment using a standardized rating approach is one of the most used methods [27]. However, challenges such as rating inconsistency across students and inconsistent clinical scenarios in practice exist. Standardized patients (SP) with skills checklists is an innovative method that can be used to assess student clinical skills. This approach provides a consistent clinical scenario, reducing variability between students’ experiences and ensuring fairness in student assessment [28].

SPs can be used beyond simulated patients by training them as examination teaching associates. In these roles, SPs can assess students based on their training and the skills checklist [29]. Given their familiarity with the cases and training as actors, SPs enable students to practice interviewing patients in a safe, simulated environment as if they are interacting with actual patients seeking care [29]. After student-SP interaction, the SP can document physical examination maneuvers performed and rate the student’s interpersonal communication, medical history, and physical examination skills.

Based on a pilot study conducted by our team, using standardized patients to assess sexual-related medical history and interpersonal communication is feasible and culturally acceptable to students [26]. However, the use of SPs in Tanzania is novel, and the rating consistency between trained SPs and faculty raters of health professional students in this context is unknown. United States-based education research suggests that while SP feedback to students can be valuable, consistency is challenging but essential in formal education settings [30]. This study aimed to evaluate the interrater reliability of ratings on sexual health-related interpersonal communication and medical history-taking between SPs and trained HCP faculty for health care professional students.

## Methods

### Study design

We conducted a cross-sectional comparative study to evaluate the interrater reliability of sexual health-related interpersonal communication and medical history-taking ratings between SPs and trained HCP faculty.

### Study setting/area

The study was conducted at the Muhimbili University of Health and Allied Sciences (MUHAS) in Dar es Salaam, Tanzania. Muhimbili University is the leading health and allied sciences university in Tanzania and trains the largest number of future health professionals. In addition to training health and allied personnel, the university has conducted key research that has been instrumental in informing Tanzanian health policy. Out of the five schools at the university, we included students from two schools; the School of Nursing and the School of Medicine.

### Study population

We recruited fourth- or third-year medical students and second- or third-year nursing students who had commenced their junior and senior clinical rotations as per the MUHAS curriculum and were accustomed to interacting with patients in practice. All students were proficient in both English (the language of instruction at MUHAS) and Kiswahili (the lingua franca in Tanzania).

### Sampling and sample size

We utilized a repeated measures ANOVA (RM-ANOVA) based on the results from a pilot study conducted in 2017. The findings from the pilot study were (𝐷=0.625, 𝑡=7.277, 𝑑𝑓=51, 𝑝≤0.01, and 𝑟=0.838. The analysis yielded 100% power with a sample size of 412 participants with a 95% confidence level and a 5% margin of error.

A stratified random sampling technique was used to recruit students from two different schools (School of Nursing and School of Medicine). This method ensures that the sample reflects the population’s diversity in terms of disciplines and year of study. Within each school, participants were asked to register via their email address on a first-come, first-served basis. After recruitment, the selected students were approached and provided with detailed information about the study. Written informed consent was obtained from those who agreed to participate before commencing any study activities.

### Standardized patient cases

Four cases were developed using an Objective Structured Clinical Examination (OSCE) format. The cases were:

1. a 37-year-old married woman with three children experiencing domestic and sexual violence from her husband. She presents a chief complaint of injury manifest by bruising throughout the body, cut wounds on the face, severe pain, severe hematoma, and swelling around her left eye.
2. a 42-year-old married man with one child. His chief complaint is being unable to maintain an erection during intercourse with his wife for about a month.
3. a 16-year-old female presents after obtaining a positive home pregnancy test. Her chief complaints are abdominal pain and vaginal bleeding. She comes to the clinic scared and unsure of her steps.
4. a 29-year-old man with a burning penile drip after having unprotected sexual intercourse with a woman he met at the club. He is also having sex with his regular sexual partner, another college student enrolled in the nursing program.

### Procedures

Twelve SPs were trained by skilled faculty to portray patients with sexual health issues. The SP training included an introduction to medical simulation, role-playing/acting within specified parameters, understanding the role of SPs in general and in individual cases, appropriate responses during interviews, and how to document feedback afterward. They were also instructed to rate their conversations with students using a standardized checklist and save the recordings to a computer server upon completion. Students were recruited through flyers on campus noticeboards and class announcements.

A total of 824 interviews (2 interviews per participants) were recorded between SPs and students. The interviews took place in a quiet, conducive room to facilitate proper interaction between the patient (SP) and the provider (student). For the conversation to be rated later, it was videotaped by the standby camera placed in front of them. The role of the research assistant (RA) was to start the video at the beginning of the interview and end it at the end. To facilitate a free interview, the RA instructed the provider(student) to wave their hand for the video to be stopped by the RA and leave the room. Once the video was stopped, the RA saved the video and prepared the camera for the next interview. Each participant had a maximum of 10 min per scenario to interview the patient before leaving for the day’s second interview. Once the interview was over, the SP had another 5 min to rate their conversation with the participant by using the standardized checklist. Participants were rated on 10 items of Interpersonal communication skills (IC) and six key items for medical history-taking skills. All responses were saved automatically in the Qualtrics database upon SP rating completion. After all, the videos were collected, they were distributed randomly to five independent healthcare professional faculty for rating using the same standardized checklist used by SPs. Each participant was rated on 10 items assessing their interpersonal communication (IC) abilities on a 3-point scale (0 = not done; 1 = partially done; 2 = done). Thus, participants could obtain a total of 20 points for each scenario. For medical history taking (MHT), six key pieces of information were identified in each case and rated on a 2-point scale. Participants received a 0 if they did not solicit this information and a 1 if they obtained it. Participants could score 0–6 for the medical history section.

### Data analysis

We present descriptive data as mean, standard deviation, frequencies, and percentages. Cohen’s kappa (κ) statistic and percent agreement, both with 95% confidence intervals (95% CIs), were used to measure inter-rater reliability between SP and HCPs [31]. As described elsewhere, κ values range between 0 (chance agreement) and 1.00 (complete agreement). Data were analyzed using R statistical software (Version 4.2.2).

## Results

### Demographic characteristics of the participants

We recruited 412 healthcare professional students from a pool of 563 eligible students, the response rate was 100%. Almost two-thirds of the participants 67% (*n* = 274), were medical students, while the remainder were nursing students. Approximately one-third of the participants, accounting for 30.6% (*n* = 126), were female, and the majority of the students, 83% (*n* = 364), identified them self as Christian by religion. The mean age of the participants was 24 years (SD = 2.46) (see Table 1). Each student interviewed two SPs with sexual health problems, and their interviews were video-recorded for rating. Consequently, each participant had two videos to be rated by both SPs and HCPs. This resulted in a total of 848 videos (with 1648 ratings) to be compared, of which there were 38% discrepancies between SP and HCP.

**Table 1:** Baseline demographic characteristics of the intervention and control groups

Characteristics	*N*	%
**Discipline**		
Medical	274	66.5
Nursing	138	33.5
**Year of Study**		
Final	201	48.8
Penultimate	211	51.2
**Gender**		
Female	126	30.6
Male	272	66
Other/Prefer Not to Answer	14	3.4
**Religious Affiliation**		
Christian	364	84
Muslim	58	14.1
Other/Prefer Not to Answer	8	1.9

#### Interpersonal communication interrater reliability between SPs and HCP

For interpersonal communication (IC), the overall median agreement between raters was slight (κ^2^ 0.0095; PA 48.9%) but it varied across cases: Erectile dysfunction (κ^2^ 0.0087; PA 46.33%), Early pregnancy (κ^2^ 0.0062; PA 46.33%), Sexual violence (κ^2^ 0.0253; PA 55.8%), and Penile drip (κ^2^ 0.00348; PA 47.3%). (see Table 2)

**Table 2:** Kappa statistics and percentage agreement for interpersonal communication in cases

Cases	PA (%)	κ	κ^2^
**Erectile dysfunction**			
Introduced them self & explained their role to SP	51	0.161	
Asked if SP had any additional concerns symptoms	35	0.092	
Treated SP respectfully	50	0.114	
Uses open-ended at first, then fills in gaps with close-ended	47.6	0.040	
Summarized and synthesized things for clarity	37.9	0.004	
Showed they were listening attentively to patient	61.7	0.081	
Expressed emotional support & care for patient	37.9	0.092	
Asked about my worries or expectations about my problem	33.5	0.051	
Used the language SP easily understood	63.6	0.118	
Provided closure with a summary and next step	45.1	0.160	
Median and IQR		0.092	**0.00874**
**Early pregnancy**			
Introduced them self & explained their role to SP	43.2	0.074	
Asked if SP had any additional concerns, symptoms	37.9	0.043	
Treated SP respectfully	50.5	0.084	
Uses open-ended at first, then fills in gaps with close-ended	37.9	-0.011	
Summarized and synthesized things for clarity	47.6	0.093	
Showed they were listening attentively to patient	53.9	0.090	
Expressed emotional support & care for patient	31.1	0.012	
Asked about my worries or expectations about my problem	51.5	0.02	
Used the language SP easily understood	63.6	0.095	
Provided closure with a summary and next step	46.1	0.168	
Median and IQR		0.079	**0.00624**
**Sexual violence.**			
Introduced them self & explained their role to SP	51.9	0.093	
Asked if SP had any additional concerns, symptoms	36.4	0.002	
Treated SP respectfully	49.5	0.096	
Uses open-ended at first, then fills in gaps with close-ended	48.1	0.026	
Summarized and synthesized things for clarity	71.8	0.085	
Showed they were listening attentively to patient	60.2	0.164	
Expressed emotional support & care for patient	47.6	0.122	
Asked about my worries or expectations about my problem	48.5	0.159	
Used the language SP easily understood	86.9	0.024	
Provided closure with a summary and next step	57.8	0.150	
Median and IQR		0.159	**0.02528**
**Penile drip**			
Introduced them self & explained their role to SP	33.5	0.041	
Asked if SP had any additional concerns, symptoms	27.2	0.031	
Treated SP respectfully	42.7	0.104	
Uses open-ended at first, then fills in gaps with close-ended	52.9	0.040	
Summarized and synthesized things for clarity	27.7	0.020	
Showed they were listening attentively to patient	66	0.116	
Expressed emotional support & care for patient	49	0.057	
Asked about my worries or expectations about my problem	42.2	0.084	
Used the language SP easily understood	82.5	0.061	
Provided closure with a summary and next step	46.6	0.176	
Median and IQR		0.059	**0.00348**

#### Medical history taking interrater reliability between SPs and HCP

Overall, the median agreement for MH was fair (κ^2^ 0.139; PA 75.02%) but it varied across cases: Erectile dysfunction (κ^2^ 0.1018; PA 70.9%), Early pregnancy (κ^2^ 0.1018; PA 74.25%), Sexual violence (κ^2^ 0.245; PA 79.2%), and Penile drip (κ^2^ 0.1109; PA 75.6%). (see Table 3)

**Table 3:** Kappa statistics and percentage agreement for medical history in cases

Cases	PA (%)	κ	κ^2^
**Erectile dysfunction**			
She is 16 years with an adult male partner	75.7	0.65	
A positive pregnancy test at home & vaginal bleeding	76.2	0.524	
Concerns about confidentiality/has a supportive aunt	87.6	0.502	
Worry about the impact on relationships at school	77.2	0.021	
In love with a boy her age	66	0.283	
Interested in contraception to prevent pregnancy	71.8	0.250	
**Median and IQR**		**0.319**	**0.10176**
**Erectile dysfunction**			
David is a 42-year-old married man	71.4	0.268	
Complains of problems maintaining erections with his wife	93.2	0.203	
Reports having male partners in the past before he married	73.8	0.467	
Started watching pornography while his wife was pregnant	68	0.263	
He and his wife have pressure for 2nd child	58.3	0.044	
David has started going to the bars and drinking heavily	60.7	0.151	
**Median and IQR**		**0.319**	**0.10176**
**Penile Drip (PD)**			
He is a single 19 years old DJ and college student.	73.3	0.253	
He presents for an evaluation of a PD & burning on urination	100	1	
He likes being with many women and having unprotected sex	66	0.326	
He is concerned about being HIV Positive.	67	0.299	
He wants STI testing	72.8	0.391	
He is concerned about losing her main partner	74.8	0.340	
**Median and IQR**		**0.333**	**0.11089**
**Sexual Violence**			
She is 30 years old, married with three children	69.9	0.253	
She is experiencing pain after being beaten by her husband	93.2	0.784	
Experiences SV, especially when the husband is intoxicated	75.2	0.476	
She is unemployed, so she does not want to report her husband	74.8	0.496	
She feels ashamed to leave because her family will not support	76.2	0.494	
She is worried and unsure about going home; it is not safe	86.4	0.529	
**Median and IQR**		**0.495**	**0.24503**

## Discussion

This study evaluated the interrater reliability of ratings on sexual health-related interpersonal communication and medical history-taking between SPs and trained HCP faculty for health care professional students. For interpersonal communication (IC), the overall median agreement between raters was slight (κ^2^ 0.0095; PA 48.9%) while the overall median agreement for medical sexual history-taking was deemed fair (κ^2^ 0.139; PA 75.02%).

Our findings with regard to interpersonal communication are inconsistent with several studies of interrater reliability between faculty/clinicians and SP [32–34]. In most of these studies’ interrater reliability ranges from fair to good, whereas our study shows slight agreement. Its noteworthy that most of these studies were conducted in high-income country settings, none specifically focused on sexual health, and all were conducted in a monolingual context. Proper sexual communication skills are essential for clinician to gather necessary information during sexual history talking [35]. It is unlikely that a clinician will be good at taking sexual history if the curriculum that they are using in their program does not emphasize sexual health. Apart from not having a sexual health curriculum, the lower agreement, may also be attributed to Besides the lack of a sexual health curriculum, the lower agreement may also be attributed to the greater complexity of the study. Given the limited use of certain technologies in low-income settings, many student participants experienced being videotaped as part of their education for the first time, making it a highly novel experience.

Linguistically, SPs predominantly used Kiswahili as they were more comfortable with it. Kiswahili, in general, has less medical jargon and fewer sexual health terms [36] During the conversation, some students were more likely to incorporate English medical terms when a proper Swahili term was unavailable, potentially limiting the SPs’ understanding. Additionally, the SPs rated students immediately after the interview without the ability to review a videotape of the interaction.

In contrast, the HCP faculty raters had the opportunity to review the tape to confirm each element under review. Therefore, the SP had to recall everything they talked about to provide a precise rating. In this situation, the SPs were likelier to check not asked than asked if they did not remember. Moreover, the rating of sexual history taking requires the rater (SP and Faculty) to judge the presence or absence of an action after listening to a long conversation between the student and SP which cannot be truly categorized as correct or incorrect but may reflect overall sentiment. For example, faculty may determine whether a student performed a communication behavior or collected relevant medical history using sentences/ language different from the checklist. While SPs may wait for the student to mention something exactly as it was written in the checklist, or use a global overall response to the student’s manner or comfort.

Regarding medical history taking, our findings align with several studies demonstrating fair to strong agreement in ratings between SP and clinician or faculty [32–34]. This consistency is unsurprising because the history of what the patient/SP is suffering from and its related details are straightforward and rated on a yes/no approach. In this way, if the SP were asked if they have a specific problem, they could easily remember and check the appropriate box. On the other hand, faculty can also check the same way based on what the student has asked the SP.

Training SPs to act as evaluators is practical and offers a unique perspective compared to faculty evaluation. If SPs were to rate the exchange by reviewing the video rather than relying on recalling, there might be higher correlations between the two types of raters.

### Limitations

This study must be considered in light of several general limitations. First, the use of formal SP is novel in Tanzania. Given the novelty of this technique, our SPs may have found it challenging to effectively assume the patient’s and student assessor’s roles. In addition, SPs rated students immediately after the interview, while faculty rated students after data collection was complete via videotaped clip. While timely assessments are essential in active learning and training environments, giving the SPs a chance to review the video may result in similar SP ratings across rater types. Lastly, our study was conducted during the COVID-19 pandemic, and our safety protocol required both SPs and students to wear masks and to maintain six-foot distance. These safety requirements might also have impacted the SP’s ability to rate the communication skills component, which relies on verbal and nonverbal communication.

### Recommendations

The medical and nursing universities in Tanzania and other low-resource settings should consider using SPs as part of health professional student education. Videotaping SP-student sessions is a valuable tool for student and faculty review; however, it may not always be feasible in real time. When considering using SPs for educational research, alignment between SPs and faculty raters should be performed to ensure the accuracy of student assessments. Reliability checks between SPs and expert raters before initiating data collection are critical for quality control. Faculty raters should be the gold standard if prior ratings cannot be harmonized. We also recommend other studies engage in additional professional development training for the SPs with some focus on the critical issues embedded in each case and expectations for the related student behaviors that coincide with each element of the assessment. Moreover, healthcare training institutions must budget for training and reimbursement when integrating SPs into their Skills Training Labs.

## Conclusion

SPs are critical for training healthcare professional students as a part of simulations in low-resource countries like Tanzania. However, to achieve a reliable and consistent rating between SPs and faculty rating, additional training for SPs and exercises to harmonize an idealized approach to rating students on critical communication and medical history-taking behaviors is warranted. In the interim, faculty ratings should be the gold standard in research and active learning environments for final assessment.

## Data Availability

The data generated in this study have been uploaded to the University of Minnesota as well as the University of Michigan repositories as per NIH data availability policy. Therefore, they are available from the corresponding author permission on reasonable request.

## Abbreviations

HCP: Health Care Providers
IC: Interpersonal Communication
MHT: Medical History Taking
MUHAS: Muhimbili University of Health and Allied Sciences
NIH: National Institute of Health
OSCE: Objective Structured Clinical Examination
RA: Research Assistant
SP: Standardized Patient
